# Socioeconomic disparities between oral cavity cancer patients in Germany

**DOI:** 10.3389/fpubh.2022.831479

**Published:** 2022-07-22

**Authors:** David Muallah, Jan Matschke, Sophie Muallah, Anna Klimova, Lysann Michaela Kroschwald, Tom Alexander Schröder, Günter Lauer, Dominik Haim

**Affiliations:** ^1^Department of Oral and Maxillofacial Surgery, Faculty of Medicine “Carl Gustav Carus”, Technische Universität Dresden, Dresden, Germany; ^2^Institute for Medical Informatics and Biometry, Technische Universität Dresden, Dresden, Germany; ^3^Center for Translational Bone, Joint and Soft Tissue Research, University Hospital “Carl Gustav Carus”, Technische Universität Dresden, Dresden, Germany

**Keywords:** oral cancer, socioeconomic factors, Germany, epidemiology, survival, level of education, oral squamous cell carcinoma, OSCC

## Abstract

**Objective:**

In many countries the access to high quality medical service depends on socioeconomic factors. Therefore, these factors are associated with the treatment and prognosis of many diseases. In Germany health care is claimed to be independent from such factors due to obligatory health insurance and a well-developed medical infrastructure. Thus, socioeconomically caused health disparities should be absent. The aim of this study was to analyze the association between socioeconomic factors and the survival of oral cavity cancer in Germany.

**Patients and methods:**

In this descriptive cohort study socioeconomic status related factors as well as demographic, tumor-specific, and comorbidity factors of 500 patients treated for oral cavity cancer were obtained in the university hospital of Dresden. Pearson correlation was used to describe associations between continuous variables. Associations between categorical variables were assessed using the chi-square test. Overall and recurrence-free survival were studied using the Kaplan-Meier method. Log-rank test was carried out to test between-group differences. Cox proportional hazard models were used to estimate the risk of death and the risk of recurrence.

**Results:**

Significant differences in overall survival were found between the different educational levels and sex. Seventy-nine percent of the patients did not have a university degree or master craftsman/craftswoman. Less discrepancy was observed according to the marital status (49.4% married/engaged vs. 47.8% single, divorced, or widowed). In the multivariable analysis only sex, age at diagnosis, the Charlson score, the number of positive lymph nodes, and the nodal status were identified as independent predictors for overall survival whereas sex and the age at diagnosis were identified as independent predictors for recurrence-free survival.

**Conclusion:**

Despite the equitable health system in Germany, significant associations between overall survival of oral cavity cancer and different socioeconomic factors could be found. For elimination of these disparities, health education programs should be established in socially deprived areas. Furthermore, clinicians should keep these factors in mind when determining recall periods for dental check-ups.

## Introduction

In 2018 355,000 new cases of oral cavity cancer were diagnosed worldwide (1). In the same year 177,000 patients died from this disease (1). In Germany nearly 5,000 new cases are diagnosed and 1,500 patients die of oral cavity cancer every year (2). Besides the low 5-year survival rate of ~42%, the tremendous limitations surviving patients suffer from make this disease a serious public health problem (3–6). The prime risk factors for oral cancer are smoking and alcohol (7). Although smoking habits in Germany show a downward trend, the incidence of oral cavity cancer stays stable. This phenomenon may be due to an increased prevalence of human papilloma virus (HPV) infections. While HPV infections were primarily considered to cause cancer of the genital sites, current studies report HPV infections to be associated with oral squamous cell carcinoma as well. Therefore, it is assumed that HPV (especially HPV16 and HPV18) may also play a role in the etiology of oral squamous cell carcinoma and should be considered as risk factor (8).

In addition to well-known risk factors there is increasing evidence that socioeconomic parameters are associated with several pathologies as well (9–14). As Bray et al. (15) could show, the incidence and mortality of oral cancer is higher in low developed countries. This circumstance suggests that this disease is associated with socioeconomic factors as well. Indeed, some studies could show a certain association between socioeconomic factors and oral cancer. Admittedly, these studies were conducted in countries with different health systems (16–21). In many health systems high quality medical care is only accessible for patients with higher socioeconomic status which could be one reason for treatment and survival differences found in these studies.

In Germany the access to medical care is primarily independent of socioeconomic factors such as marital status, sex, education, and income. Therefore, the treatment and mortality of oral cancer should be equal in different social stratums. Nevertheless, previously conducted studies report inconsistent data (22, 23). While Klingelhöffer et al. (23) could not find survival differences between different occupational stratums, Finke et al. (22) reported clear gradients across area-based socioeconomic deprivation quintiles. To investigate whether the socioeconomic status is associated with survival of oral cavity cancer in Germany, we conducted a retrospective study with 500 patients that were treated for oral cancer in our clinic, a head and neck cancer center in Saxony/East Germany, in the period from 2013 to 2019.

## Patients and methods

### Patient data

A chronological list of all patients that applied to our clinic for tumor treatment or follow up between 2013 and 2019 was screened for the ICD Codes C00–C06 (cancer of the oral cavity). Out of these patients the first 500 were included in the study and follow up data were obtained retrospectively. A positive vote of the local ethics commission was received (IRB00001473, BO-EK-415092020). Following parameters were used as socioeconomic status related factors after chart review: Level of education (university degree or master craftsman/craftswoman vs. others or unknown level of education), sex (male vs. female), marital status (married vs. single/divorced/widowed/unknown), and unemployment rate of the neighborhood. We also included the distance to the clinic (continuous variable) as an additional parameter since it is considered in different previous studies (24, 25). The distance to the clinic was calculated as the distance of the patients' post code to our institution using Google maps. The unemployment rate of the neighborhood was approximately estimated from the statistics of the federal agency for work and the demographic statistics of the federal states by dividing the number of unemployed inhabitants by the number of inhabitants aged between 18 and 65 in each neighborhood.

Following demographic, tumor-specific and comorbidity factors were obtained *via* chart review of the anamnesis documents, the preoperative cardiopulmonary risk assessment and the hospital information system: age at time of diagnosis (continuous), body-mass-index (underweight vs. normal weight vs. overweight for the bivariate analysis and as a continuous variable for multivariable analysis), smoking status (non-smokers vs. current smokers/former smokers), alcohol intake (no intake vs. current intake or former intake), Charlson score (continuous), local tumor stage (ordinal following the TNM classification), lymph node status (ordinal following the TNM classification), number of positive dissected lymph nodes (continuous), adjuvant chemotherapy (yes vs. no/unknown), adjuvant radiotherapy (yes vs. no/unknown), recurrence-free survival and overall survival (continuous). As recurrence, all neoplasms that were locally or histologically related to the previous tumor were counted. The socioeconomic status related factors as well as demographic and comorbidity factors reflect the status at time of diagnosis. The follow up started after first-line therapy for the primary tumor and was carried out by specialists in our clinic. First-line treatment was always surgery if possible. For more severe cases or recurrences chemoradiotherapy was administered as suggested in the German guideline for diagnosis and therapy of oral cavity tumors (26).

### Statistical analysis

The statistical analysis was carried out using SPSS Statistics 26 (IBM, Armonk, New York, USA). For continuous variables, the observed mean and range are reported. Categorical variables are summarized using absolute and relative frequencies. The Pearson correlation was used to describe the association between continuous variables. The association between categorical variables was assessed using the chi-square test. Survival rates calculation (overall survival and recurrence-free survival) was done using the Kaplan-Meier method, and log-rank test was carried out to test between-group differences. Finally, a multivariable survival analysis was performed using a Cox proportional hazards model for risk of death (overall survival) and the risk of recurrence. Therefore, parameters were chosen based on previous literature and results of the univariate analysis. Hazard ratios (HR) were calculated for each parameter. For statistical inference, the significance level of 5% (two-sided) was assumed. Patients with missing data were censored.

## Results

Out of the 500 included patients 194 died in the observation period, which leaves 306 patients that were censored. The mean age at time of diagnosis was 61.49 years (Table 1). 66.8% of the study population were male and 64.2% of the patients were either smokers or former smokers. With 79% the majority of the patients did not have an university degree or master craftsman/craftswoman. Less discrepancy was observed according to the marital status. While 49.4% of the patients were either married or engaged, 47.8% were single, divorced, or widowed. Also alcohol intake was nearly equal as 53.6% stated to drink or had drunk and 46.4% of the patients claimed to not drink alcohol. Only 40.4% were diagnosed with a local tumor stage of pT3 or pT4 and nearly half of the patients (45.6%) had a positive lymph status. The mean unemployment rate of the neighborhood was calculated as 5.13% and the mean distance to the clinic was 32.82 km. A mean overall survival of 9.8 years could be estimated whereas the recurrence-free survival was much shorter with a mean of 2.2 years. The mean follow-up was 4 years (Table 1).

**Table 1:** Demographic data of the study population.

**Parameter**		***n*** **= 500**	**Proportion (%)**
Deaths (Overall)		194	38.8
Censored patients		306	61.2
**Sex**			
Female patients		166	33.2
Male patients		334	66.8
**Level of education**			
University degree or master craftsman/craftswoman		51	10.2
No university degree or master craftsman/craftswoman		395	79.0
Unknown level of education		54	10.8
**Smoking status**			
Current/Former smokers		321	64.2
Non-smokers		179	35.8
**Alcohol status**			
Current/Former alcohol intake		268	53.6
No alcohol intake		232	46.4
**Marital status**			
Married patients		247	49.4
Single/widowed/divorced patients		239	47.8
Unknown marital status		14	2.8
**TNM classification**			
T-stage > 2		202	40.4
N-stage > 0		180	45.6
Adjuvant chemotherapy		59	11.8
Adjuvant radiotherapy		202	40.4
	**Minimum**	**Maximum**	**Mean**
Unemployment rate of neighborhood	1.1	15.5	5.13
Distance to clinic (km)	0.68	587.55	32.82
Body mass index	16	45	25.52
Age at diagnosis	14	90	61.49
Charlson score	0	12	3.85
Positive lymph nodes	0	36	1.11
Removed lymph nodes	0	88	23.60
Overall survival (months)	0	217	43.25
Recurrence-free survival (months)	1	127	24.58
Follow-up (months)	0	217	48.25

**Figure 1:**
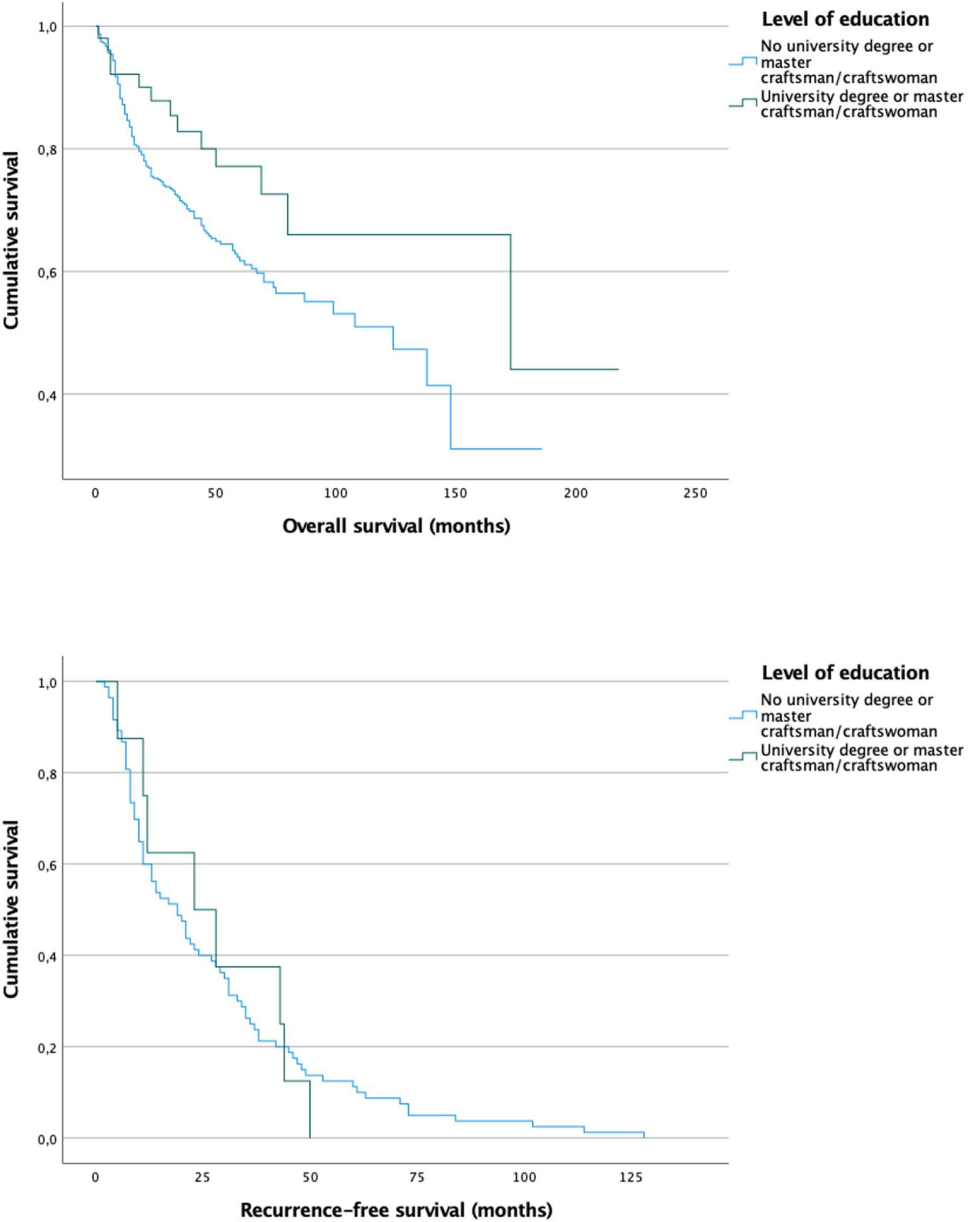
Survival curves for patients with (mean overall survival 12.3 years) and without (mean overall survival 8.6 years) university degree or craftsmen/craftswoman show a significant difference in overall survival (*p* = 0.039) but not in recurrence-free survival (*p* = 0.99).

For survival analysis the Log Rank test was applied to the nominal socioeconomic status related factors both for overall survival and for recurrence-free survival. As shown in Figure 1 a clear difference in overall survival could be observed between the two levels of education (Figure 1). While patients with a higher level of education had a mean overall survival of 12.3 years, patients with a lower level of education survived only 8.6 years (*p* = 0.039). Nevertheless, no significant difference could be identified in recurrence-free survival.

**Figure 2:**
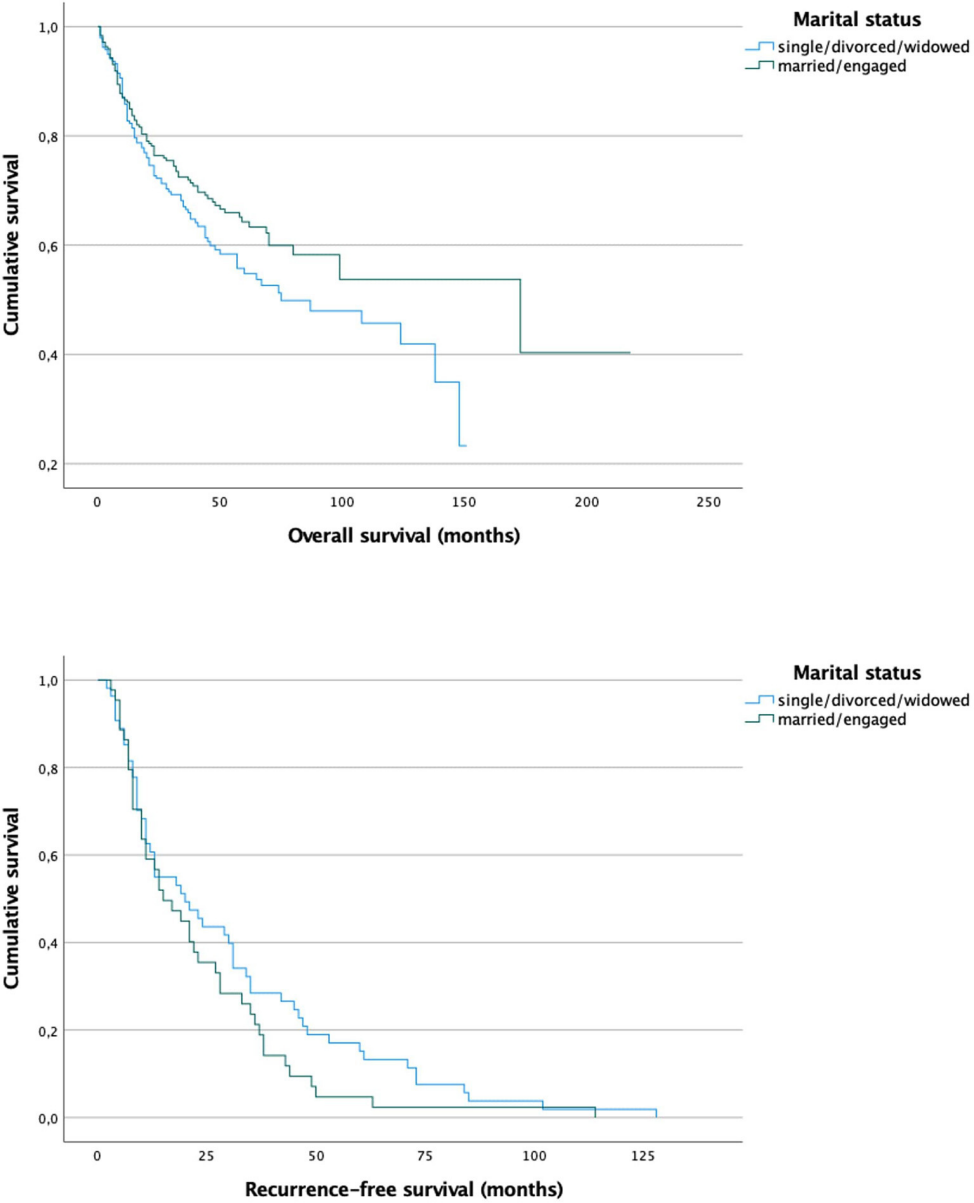
Survival curves for patients that are married/engaged (mean overall survival 10.6 years) and patients who are single/divorced/widowed (mean overall survival 7 years) show a difference, which slightly misses significance for overall survival (*p* = 0.068) but seems not to be significant for recurrence-free survival (*p* = 0.164).

Studying the survival after stratifying into different marital statuses revealed a longer overall survival for married/engaged patients (10.6 vs. 7 years mean), which slightly missed significance (*p* = 0.068). For recurrence-free survival no significant divergence between the survival curves was observed (Figure 2).

**Figure 3:**
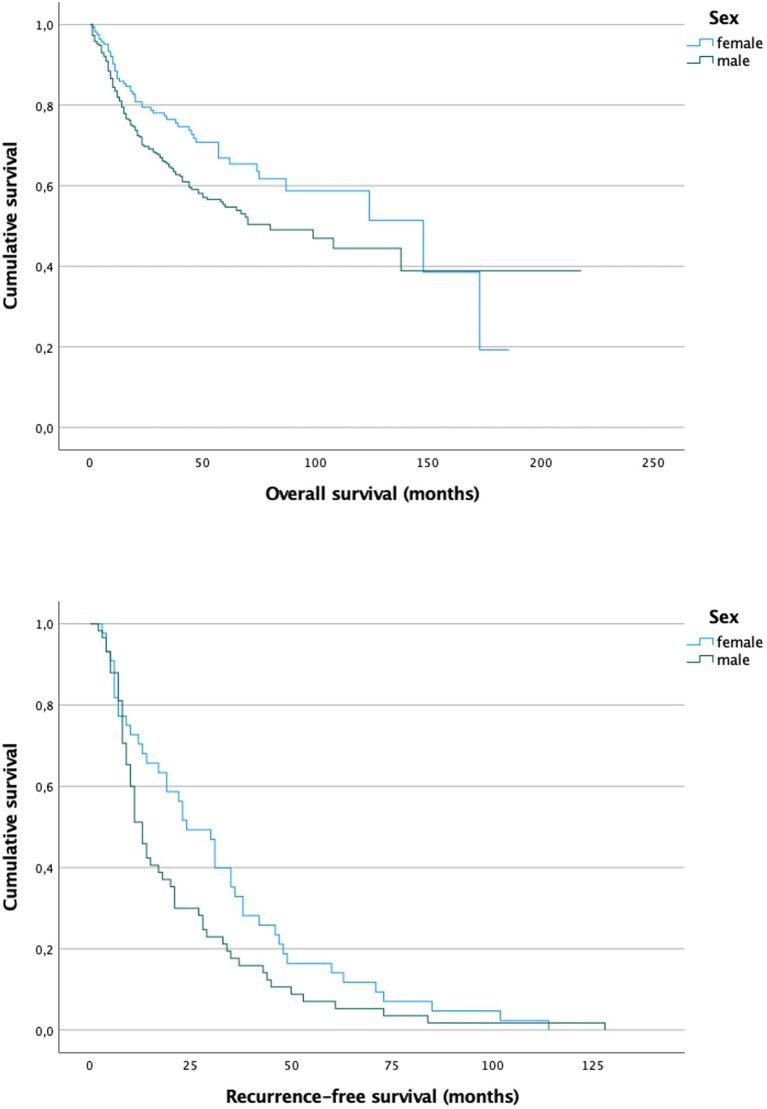
Survival curves for male patients (mean overall survival 9.2 years) and female patients (mean overall survival 9.2 years) show a slight but significant difference in overall survival (*p* = 0.016), while recurrence-free survival slightly misses significance (*p* = 0.068).

As another socioeconomic status related factor, the sex was observed for survival differences (Figure 3). As shown in Figure 3, women had a minimally longer overall survival (*p* = 0.016) while no significant difference was observed in recurrence-free survival (0.068). Nevertheless, the Kaplan Meier curve shows, that male patients tend to have earlier recurrence as the curve drops faster.

To identify possible causes of the survival differences observed in the Kaplan–Meier curves, the socioeconomic factors were analyzed in cross tabs together with variables that are known to have an impact on health and survival in general.

The bivariate analysis of the level of education revealed a significantly lower chance of nicotine absence (OR = 0.27, *p* < 0.001) and alcohol absence (OR = 0.3, *p* < 0.001) in the group of patients with lower level of education (Table 2). Furthermore, these patients are less likely to be diagnosed with an early tumor stage (OR = 0.44, *p* = 0.016) and to be treated without adjuvant chemotherapy (OR = 0.13, *p* = 0.02).

**Table 2:** Crosstab and chi square test for level of education and other nominal parameters that are associated with survival.

**Variable**	**No UD/MC**	**UD/MC**	**OR (*p*)**
Non-smoker	132 (33.4%)	33 (64.7%)	0.27 (<0.001)
pT <3	228 (59.9%)	39 (76.5%)	0.44 (0.016)
No alcohol intake	175 (44.3%)	37 (72.5%)	0.3 (<0.001)
No adjuvant Cx	342 (86.6%)	50 (98%)	0.13 (0.02)

*UD/MC, university degree or master craftsman/craftswoman; OR, odds ratio*.

Also, the marital status showed significant differences according to smoking status, the local tumor stage, alcohol intake and the BMI (Table 3). Single, divorced, and widowed patients are less likely to be non-smokers (OR = 0.562, *p* = 0.002), have fewer chances to be diagnosed with tumor stages pT1, pT2, or Tis (OR = 0.6, *p* = 0.007) and less frequently abstain from alcohol (OR = 0.68, *p* = 0.034). Furthermore, the BMI seems to be significantly associated with the marital status as married and engaged patients are more likely to be overweight while single, divorced, and widowed people are more likely underweight (*p* < 0,001).

**Table 3:** Crosstab and chi square test for marital status and other nominal parameters that are associated with survival.

**Variable**	**Single/divorced/widowed**	**Married/engaged**	**OR (*p*)**
Non-smoker	71 (29.7%)	106 (42.9%)	0.56 (0.002)
pT <3	123 (52.8%)	158 (65%)	0.6 (0.007)
No alcohol intake	100 (41.8%)	127 (51.4%)	0.68 (0.034)

*OR, odds ratio*.

Similar associations were observed when analyzing sex (Table 4). Female patients were 5.1 times more likely non-smokers (OR = 5.1, *p* < 0.001), had a nearly two times higher chance to be diagnosed with lower tumor stages (OR = 1.95, *p* < 0.001), do more often abstain from alcohol (OR = 5.9, *p* < 0.001) and have a higher chance to be treated without adjuvant chemotherapy (OR = 2.69, *p* = 0.005).

**Table 4:** Crosstab and chi square test for sex and other nominal parameters that are associated with survival.

**Variable**	**Male**	**Female**	**OR (*p*)**
Non-smoker	78 (23.4%)	101 (60.8%)	5.1 (<0.001)
pT <3	173 (53.4%)	114 (69.1%)	1.95 (<0.001)
No alcohol intake	109 (32.6%)	123 (74.1%)	5.9 (<0.001)
No adjuvant Cx	284 (85.3%)	156 (94%)	2.69 (0.005)

*OR, odds ratio*.

With 38.2% the floor of the mouth was the most frequent tumor site followed by the tongue (26.4%) and the lower alveolar ridge (16.6%). However, no significant correlations with socioeconomic factors could be found.

The socioeconomic status related factors were also integrated in a multivariable Cox proportional hazard model together with general tumor and comorbidity parameters, which were suspected to affect the risk of death from oral cancer. Table 5 shows the results of the multivariable analysis calculated for overall survival. Only sex (male vs. female, HR = 1.69, 95% CI = 1.05–2.73), age at diagnosis (HR = 1.03, 95% CI = 1–1.06), Charlson score (HR = 1.12, 95% CI = 1.03–1.22), the lymph node status (HR = 1.31, 95% CI = 1.15–1.49), and the number of dissected positive lymph nodes were identified to have significant impact on overall survival (Table 5). The BMI (HR = 0.96, 95% CI = 0.92–1) slightly missed significance with a *p*-value of 0.053.

**Table 5:** Cox proportional hazard model for overall survival.

**Parameter**	**HR**	**95% CI**	***p***
Sex male (vs. female)	1.69	1.05–2.73	0.03
Marital status married/engaged (vs. single/divorced/widowed)	0.85	0.55–1.31	0.451
University degree or master craftsman/craftswoman (vs. no University degree or master craftsman/craftswoman)	0.67	0.32–1.4	0.283
Distance to clinic (continuous variable, per km)	1	0.99–1	0.487
Unemployment rate of neighborhood (continuous variable, every full %)	1	0.93–1.08	0.957
Age at diagnosis (continuous variable, per year)	1.03	1–1.06	0.025
Charlson score (continuous variable, per point)	1.12	1.03–1.22	0.012
BMI (continuous variable, per point)	0.96	0.92–1	0.053
pT (categorial variable, per ascending stage)	1.15	0.96–1.36	0.129
pN (categorial variable, per ascending stage)	1.31	1.15–1.49	<0.001
Positive LN (continuous, per positive lymph node)	1.1	1.06–1.15	<0.001

Same variables were used for multivariable analysis of recurrence-free survival. As shown in Table 6 the sex (HR = 2.14, 95% CI = 1.01–4.53) and the age at diagnosis (HR = 1.05, 95% CI = 1.01–1.09) were identified as independent predictors of recurrence-free survival (Table 6).

**Table 6:** Cox proportional hazard model for recurrence-free survival.

**Parameter**	**HR**	**95% CI**	***p***
Sex male (vs. female)	2.14	1.01–4.53	0.047
Marital status married/engaged (vs. single/divorced/widowed)	1.49	0.81–2.74	0.198
University degree or master craftsman/craftswoman (vs. no University degree or master craftsman/craftswoman)	0.67	0.23–1.9	0.448
Distance to clinic (continuous variable, per km)	1	0.99–1	0.391
Unemployment rate of neighborhood (continuous variable, every full %)	0.99	0.92–1.08	0.878
Age at diagnosis (continuous variable, per year)	1.05	1.01–1.09	0.021
Charlson score (continuous variable, per point)	0.88	0.74–1.05	0.147
BMI (continuous variable, per point)	1.02	0.95–1.08	0.637
pT (categorial variable, per ascending stage)	1.14	0.86–1.51	0.365
pN (categorial variable, per ascending stage)	1.05	0.82–1.33	0.727
Positive LN (continuous, per positive lymph node)	1.05	0.87–1.27	0.617

## Discussion

In many countries of the world the access to high quality medical service depends on socioeconomic factors such as income, level of education, the medical infrastructure of the neighborhood and even sex. For this reason the course of many diseases was found associated with socioeconomic factors (9–14). One of those is oral cancer (15–22, 27–31). In Germany health care is claimed to be independend from such factors due to obligatory health insurance and a well-developed medical infrastructure. Therefore, the treatment and survival of oral cavity cancer should be independent from socioeconomic parameters in Germany. Despite this hypothesis, in this study we found significant associations between the risk of death from oral cancer and different socioeconomic factors such as sex, marital status and the educational level.

As shown in our study, more men suffer from oral cancer than women (66.8 vs. 33.2%). This has also been found in other studies (32). It is believed, the main reason for this is due to the fact that men tend to expose themselves more frequently to risk factors, specifically nicotine and excessive alcohol intake, which are prime risk factors for oral cancer (33). This assumption could be supported by our findings, that women were less likely to either smoke or consume alcohol (Table 4). Women were also more likely to be diagnosed with an early tumor stage, which correlates with a better prognosis, compared to more advanced stages. Interestingly, our results also revealed that women had a greater chance to not need adjuvant chemotherapy, which could be interpreted as another surrogate for a less sever disease in women since adjuvant chemotherapy is only administered for advanced tumor stages. These findings result in women tending to live longer than men after the tumor diagnosis (*p* = 0.016). Longer survival of woman was also observed previously. In a study of Listl et al. (34), 5-year survival rates of woman and men were reported with 61.3 and 53.0%, respectively. Nevertheless, no significant difference regarding the recurrence-free survival could be found (*p* = 0.068). According to the other parameters (less smoking and drinking, earlier diagnosis etc.) a longer recurrence-free survival could have been expected. However, although the significance is slightly missed, a trend for a longer recurrence-free survival in women can be seen indeed. Unfortunately, our study cannot assess sex differences at molecular or genetic level. Though, it was previously shown that sex specific hormones can have an impact on other tumor entities such as bladder cancer (35–37). Whether these findings also account for oral cancer remains unclear and should be investigated in further studies.

The level of education is commonly used as a proxy for the socioeconomic status (9, 10, 12–14, 27, 38, 39). We could find, that only 10.2% of our patients had an university degree or a master craftsman/-women, while 79.0% did not have either of them. In contrast to our study sample, the ratio of people with university degree is much higher in the total German population. According to the Federal Statistical Office of Germany 18.5% of the German population have an university degree (40). In Dresden (28.54%) as well as in Saxony (14.92%) the ratio of people with university degree differs from the German average but is still higher compared to our study population (40). Therefore, it could be assumed that the educational level correlates with the incidence of oral cavity cancer. There are several sources, which state that higher levels of education positively correlate with health and the absence of severe illnesses. The main reason for this may be a lower consumption of pro-cancerogenic products such as cigarettes, alcohol and certain diets (39, 41). The findings of our study substantiate this hypothesis as the risk of smoking and drinking alcohol was significantly higher in patients of lower educational level with a *p* < 0.001, respectively. Besides a high association of oral cavity cancer and a lower educational level, we also found a significantly shorter overall survival of these patients (Figure 1, *p* = 0.039). A reason could be that people of lower education have less health awareness and knowledge. Azimi et al. (42) conducted a survey with 1,312 Iranian inhabitants of different socioeconomic levels. The questionnaire tested the knowledge of symptoms and risk factors of oral cancer. As expected, people of lower educational levels had a minor knowledge of oral cancer. Similar results were observed in the USA (43). Also in Germany a divergency in awareness about oral cancer between different socioeconomic stratums was reported. In a survey with 1,000 participants from North Germany people with low income, elementary school education and blue collar workers had less knowledge about diagnosis and risk factors of oral cancer (44). Therefore, it could be assumed that these people are less cautious and early symptoms remain unnoticed. This would also explain our findings that people of lower education are more frequently diagnosed with higher tumor stages compared to people with higher education (Table 2). Surprisingly, Lins et al. (45) reported reverse findings in a cohort of 51,116 patients in brazil. A higher number of advanced cancer diagnoses was found in the group of higher education. Nevertheless, it is noteworthy that 25.8% of their study population appeared to have inaccurate data about the educational status. Additionally, 58.9% of their sample had an educational level of elementary or middle school while only 2.6% stated to have a college degree, which could cause bias in the analysis. Interestingly, we could not find a significant difference in recurrence-free survival between both educational groups. The reason for this could be the high-quality medical care every German resident can claim due to the German medical insurance system. The quality and the expand of the medical provision are independent of any socioeconomic parameters. Every patient is introduced to the same treatment and follow-up care.

**Figure 4:**
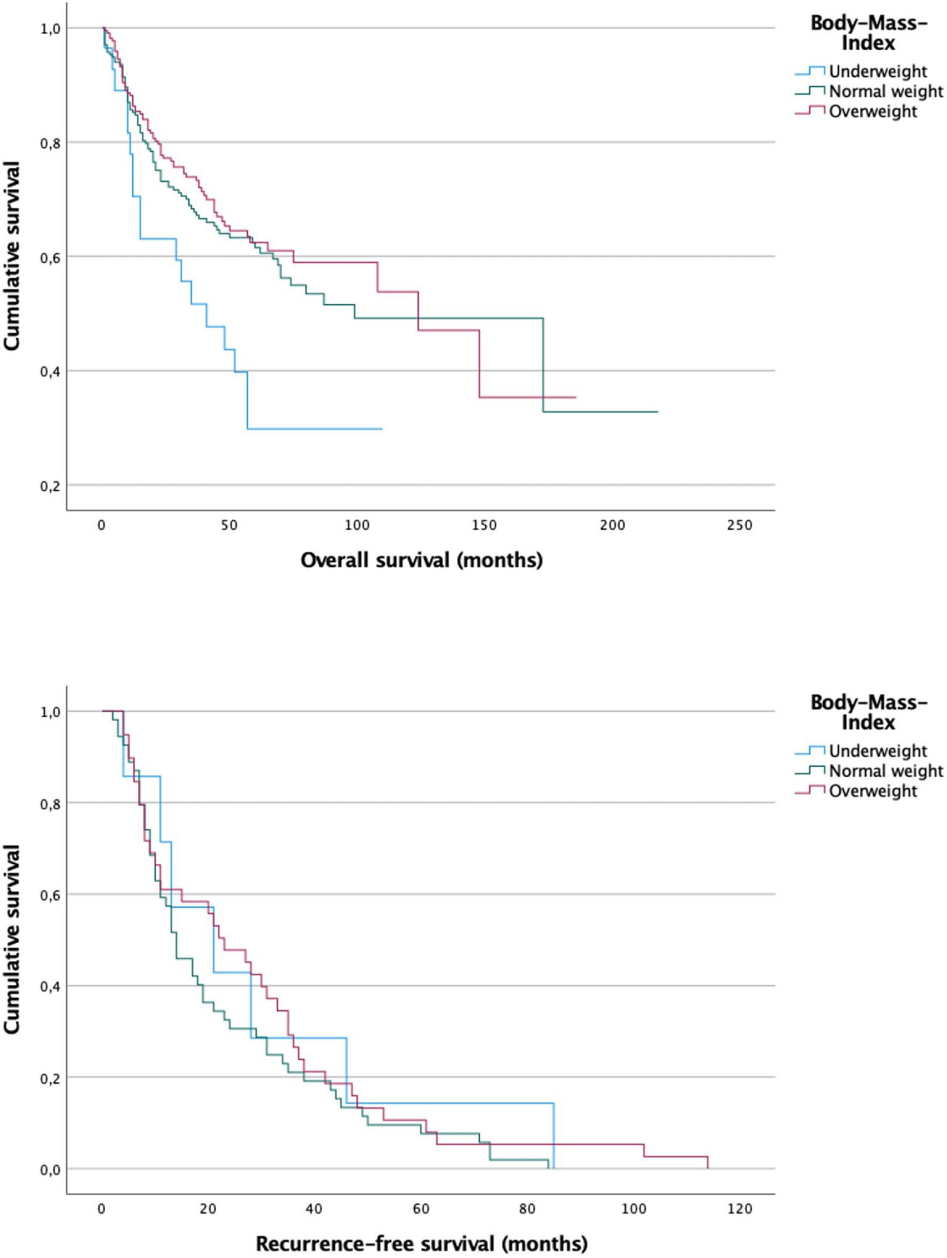
Survival curves for underweight, normal weight and overweight patients. A significant difference could be observed between underweight patients compared to the other groups in overall survival (*p* = 0.012). While underweight patients had a mean overall survival of 4.2 years normal weight and overweight patients had a mean overall survival of 9.7 and 9 years, respectively. There was no significant difference in recurrence-free survival between the three groups (*p* = 0.482).

We furthermore investigated the marital status as another socioeconomic factor. According to our findings, the marital status seems to be associated with tobacco and alcohol intake and also the tumor stage at diagnosis. It is well-known that people in relationships are less likely to be addicted to either smoking or alcohol (46–49). Therefore, they are less frequently exposed to the typical risk factors of oral cancer. In addition, we observed that married patients tend to be diagnosed with earlier tumor stages. Probably, these findings are due to the fact, that people in a relationship take care of each other and motivate their partners to see a doctor early when something is wrong. This phenomenon has also been reported for other tumor entities (38, 50). However, we could only find a trend for better overall survival of married patients, which slightly missed significance (*p* = 0.068). Similar findings were reported by Klingelhöffer et al. (23) who analyzed data of 400 patients in Southern Germany and found higher 5-year survival rates for married patients although not significant (70.8 vs. 53.7%; *p* = 0.084). The BMI, on the other hand, was found to be higher in married patients (p = 0.001), while more single/divorced/widowed patients were underweight. The weight plays an important role in the overall survival. Our results suggest that underweight patients have a significantly shorter overall survival compared to normal and overweight patients (Figure 4; 4.2 vs. 9/9.7 years; *p* = 0.012). Underweight patients have less reserves to withstand a malignant illness with an exhausting surgical procedure in the beginning, followed by a long postoperative rehabilitation. Especially in oral cancer the eating and swallowing is compromised in many cases so that deficiency syndromes can evolve. In underweight patients these syndromes may worsen the overall survival. The recurrence-free survival, however, was not influenced by the BMI.

Taking into consideration, that married patients seem to have better physical conditions, which in turn is associated with a better overall survival, it could be assumed, that married patients have a better prognosis after diagnosis of oral cancer. Nevertheless, in our sample the overall survival was not significantly different compared to single/divorced/widowed patients (10.6 vs. 7 years, *p* = 0.068), but a trend for a longer overall survival of married patients can be seen.

Finally, we also performed Cox proportional hazard regression to find out, whether the socioeconomic related factors could be identified as independent predictors for risk of recurrence and overall survival risk of death. In this analysis, besides tumor specific parameters (nodal stage, number of positive lymph nodes) and general comorbidity parameters (age, Charlson score), the sex was the only socioeconomic status related parameter which was identified as an independent predictor for both overall (HR 1.69, 95% CI 1.05–2.73, *p* = 0.03) and recurrence-free survival (HR 2.14, 95% CI 1.01–4.53, *p* = 0.047). Other analyzed socioeconomic parameters, such as the educational level, the marital status, or the unemployment rate of the neighborhood did not have a significant influence on the overall and recurrence-free survival. Also the distance to the clinic did not show a significant correlation. Similar findings were reported by Radespiel-Tröger et al. who investigated cancer incidence on the level of districts in Bavaria. They reported a correlation between oral cancer incidence and population density. However, after multivariable adjustment the district type could not be identified as an independent risk factor (51). Especially the missing significance of the distance to the clinic is another surrogate for the high quality of the German health system. In other countries the medical infrastructure and the public transport is less developed which causes difficulties for patients to see a doctor, especially if they cannot afford an own car. An American study could show that the travel time to the medical provider was positively correlated with the diagnosed tumor stage (24). Farquhar et al. reported driving times of more than 1 h. Similar findings were reported from an Australian study group (25).

As our study is of retrospective nature, some limitations must be mentioned. All the obtained data only show correlation but no causality. Therefore, a causal connection between the different factors seems to be very likely and plausible but cannot eventually be proved. Another limitation is the lack of data regarding some further suspected causalities. One example is the difference in the overall survival of women and men and the possible influence of sex specific molecular mechanisms, which are already proved for other tumor entities such as bladder cancer (21–23). One more limitation is the missing HPV status of our patients. HPV infection is an increasing cause for oral squamous cell carcinoma (8). The HPV infection can be avoided by vaccination as well as appropriate sexual hygiene, which are both educational issues. Therefore, oral cancer caused by HPV may also show associations with socioeconomic factors and should be investigated in further studies. In our study, significance was slightly missed for some correlations such as the overall survival of married patients (*p* = 0.068) and the recurrence free survival of women (*p* = 0.0.68). Therefore, a bigger sample of patients would maybe bring more clarity and should be the asset of further investigations. Additionally, our cohort is hospital based, which can lead to bias as our clinic is a certified head and neck cancer center. Therefore, the cases of our clinic may be more complex and severe compared to other clinics. This affects the cohort as well as the treatment outcome. A better approach would be to perform multicenter studies to confirm our findings. This approach would also eliminate the selection bias which is caused by the regional limitation of our study (only patients from Saxony/East Germany).

Socioeconomic status related factors seem to be associated with survival of oral cancer in unadjusted analysis. Not only was the majority of our sample found to have a lower socioeconomic status. A higher level of education is associated with a longer overall survival. However, after adjusting for clinical prognostic (and comorbidity) factors the socioeconomic parameters were no longer associated with survival. We believe these findings are caused by carcinogenic habits such as smoking or drinking, which are significantly more frequent in patients with lower socioeconomic status. To face and alleviate these disparities, more health education for certain target groups is needed. Furthermore, clinicians should consider socioeconomic factors when determining recall periods for dental check-ups.

## Data availability statement

The original contributions presented in the study are included in the article/supplementary material, further inquiries can be directed to the corresponding author/s.

## Ethics statement

The studies involving human participants were reviewed and approved by Ethics Committee of the Technical University Dresden. Written informed consent from the participants' legal guardian/next of kin was not required to participate in this study in accordance with the national legislation and the institutional requirements.

## Author contributions

This study was conceptualized by DM, DH, and GL. SM and DM contributed to collecting data. Analyzing data was carried out by DM and AK. Drafting of the manuscript was done by DM and JM. SM, AK, LK, TS, GL, and DH contributed to revising and approving the final version of the manuscript. All authors contributed to the article and approved the submitted version.

## Conflict of interest

The authors declare that the research was conducted in the absence of any commercial or financial relationships that could be construed as a potential conflict of interest.

## Publisher's note

All claims expressed in this article are solely those of the authors and do not necessarily represent those of their affiliated organizations, or those of the publisher, the editors and the reviewers. Any product that may be evaluated in this article, or claim that may be made by its manufacturer, is not guaranteed or endorsed by the publisher.
